# Coherently controlled quantum features in a coupled interferometric scheme

**DOI:** 10.1038/s41598-021-90668-8

**Published:** 2021-05-27

**Authors:** Byoung S. Ham

**Affiliations:** Center for Photon Information Processing, School of Electrical Engineering and Computer Science, Gwangju Institute of Science and Technology, 123 Chumdangwagi-ro, Buk-gu, Gwangju, 61005 South Korea

**Keywords:** Quantum physics, Quantum optics

## Abstract

Over the last several decades, entangled photon pairs generated by spontaneous parametric down conversion processes in both second-order and third-order nonlinear optical materials have been intensively studied for various quantum features such as Bell inequality violation and anticorrelation. In an interferometric scheme, anticorrelation results from photon bunching based on randomness when entangled photon pairs coincidently impinge on a beam splitter. Compared with post-measurement-based probabilistic confirmation, a coherence version has been recently proposed using the wave nature of photons. Here, the origin of quantum features in a coupled interferometric scheme is investigated using pure coherence optics. In addition, a deterministic method of entangled photon-pair generation is proposed for on-demand coherence control of quantum processing.

## Introduction

Quantum entanglement^1^ is the heart of quantum technologies such as quantum computing^2^, quantum communications^3–5^, and quantum sensing^6,7^. Although intensive research has been performed in both interferometric and noninterferometric schemes for quantum features such as the Hong–Ou–Mandel (HOM) dip^8–10^, photonic de Broglie wavelength (PBW)^11–13^, Bell inequality violation^14–16^, and Franson-type nonlocal correlation^17–19^, the fundamental physics of entangled photon-pair generation itself has still been vailed in terms of probabilistic measurements via coincidence detection of coupled photon pairs. Thus, nondeterministic measurement-based quantum technologies have prevailed, resulting in extreme inefficiency compared with their classical counterparts that are deterministic and macroscopic.

Recently, a novel method of deterministic quantum correlation has been proposed and demonstrated to unveil secretes of quantum entanglement for both HOM dip and PBW using the wave nature of photons^20–23^. The HOM-type correlation is due to photon bunching on a BS via destructive quantum interference between paired entangled photons, while PBW is due to higher order entangled photons such as a N00N state in a Mach–Zehnder interferometer. As a result, the fundamental physics of quantum features has been found in the phase property of a coupled system, where the coupled system does not have to be confined by the Heisenberg’s uncertainty principle. Based on this wave nature of photons, collective control of coherent photons is a great benefit for macroscopic quantum technologies compatible with the classical counterparts. Here, the fundamental physics of quantum correlation is investigated using the wave nature of photons to identify the origin of quantum features demonstrated in an interferometric scheme^24^. For typical $\chi^{\left( 2 \right)}$-generated entangled photon pairs, some misunderstandings regarding quantum correlation are pointed out not to criticize but to support the novelty of the wave nature of photons. Without violating quantum mechanics, a proper choice of photon property should depend on photon resources according to the wave-particle duality^25^. Finally, a coherence version of quantum feature generation is proposed for potential applications of deterministic and macroscopic quantum information processing.

**Figure 1:**
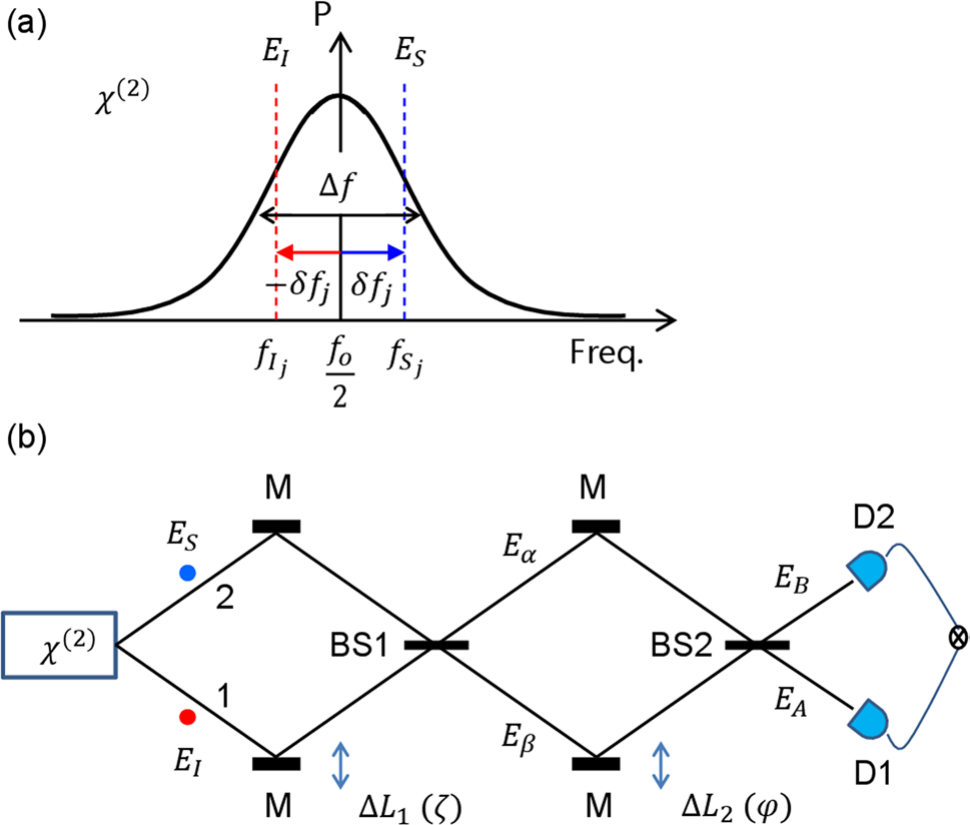
Interferometric quantum feature generation. (**a**) A SPDC-based photon-pair bandwidth. (**b**) A SPDC-based coupled interferometric scheme. *BS* Beam splitter, *D* Detector, *M* Mirror. $\zeta = \frac{2\pi }{\lambda }\Delta L_{1}$; $\varphi = \frac{2\pi }{\lambda }\Delta L_{2}$; Δ*f*: bandwidth; $\delta f_{j}$: random symmetric detuning of the jth entangled photon pair.

Figure 1 shows a particular scheme of HOM-type quantum correlation in a coupled interferometric scheme, where entangled photon pairs are generated from spontaneous parametric down conversion (SPDC) processes in a $\chi^{\left( 2 \right)}$ nonlinear material^24^. Due to the spontaneous emission decay process, an initial phase is randomly assigned to each photon pair, where each photon pair has also a random frequency detuning from the fixed half-frequency of the pump photon used for $\chi^{\left( 2 \right)}$. To satisfy the energy conservation law in the $\chi^{\left( 2 \right)}$ process, the random detuning of the photon pairs must be symmetric as shown in Fig. 1a. In related HOM-type experiments, a typical line shape observed by coincidence measurements shows a broad dip, whose decay is the inverse of the photon pairs’ bandwidth ${\Delta }f$. Unlike the theory in Ref.^20^ based on the wave nature of photons, however, the λ-dependent $g^{\left( 1 \right)}$ correlation has never been observed, where the $g^{\left( 1 \right)}$ correlation is a typical double-slit interference fringe. In the present paper, both reasons for the missing $g^{\left( 1 \right)}$ correlation in a single MZI and the revival of the $g^{\left( 1 \right)}$ correlation in a doubly-coupled MZI are investigated to unveil the physical origin of quantum features.

## Results

For an analytic discussion as to why there is no $g^{\left( 1 \right)}$ correlation in a HOM dip, a typical SPDC-generated entangled-photon system is used as shown in Fig. 1, where $E_{S}$ and $E_{I}$ represent the SPDC-generated signal and idler photon pair, respectively. Assuming there is a specific phase relation between the paired photons, signal $\left( {E_{S} } \right)$ and idler $\left( {E_{I} } \right)$^20^, the basic equations for coincidence detection measurements can be derived using general matrix representations of pure coherence optics for Fig. 1b, where $$\left[ {\begin{array}{*{20}c} {E_{\alpha } } \\ {E_{\beta } } \\ \end{array} } \right] = \left[ {BS1} \right]\left[\upzeta \right]\left[ {\begin{array}{*{20}c} {E_{I} } \\ {E_{S} } \\ \end{array} } \right]$$ and $$\left[ {\begin{array}{*{20}c} {E_{A} } \\ {E_{B} } \\ \end{array} } \right] = \left[ {BS2} \right]\left[ \upvarphi \right]\left[ {BS1} \right]\left[ \upzeta \right]\left[ {\begin{array}{*{20}c} {E_{I} } \\ {E_{S} } \\ \end{array} } \right]$$, $$\left[ {BS2} \right] = \left[ {BS1} \right] = \frac{1}{\sqrt 2 }\left[ {\begin{array}{*{20}c} 1 & i \\ i & 1 \\ \end{array} } \right]$$, $$\left[\upzeta \right] = \left[ {\begin{array}{*{20}c} {e^{i\zeta } } & 0 \\ 0 & 1 \\ \end{array} } \right]$$, and $$\left[ \upvarphi \right] = \left[ {\begin{array}{*{20}c} 1 & 0 \\ 0 & {e^{i\varphi } } \\ \end{array} } \right]$$^21^. Here, introduction of coherence optics is a choice matter without violation of quantum mechanics^25^. The jth input photon pair $E_{{S_{j} }}$ and $E_{{I_{j} }}$ can be described with the wave nature property, where $E_{{S_{j} }} = E_{0} e^{{i\left( {k_{{S_{j} }} r - 2\pi f_{{S_{j} }} t + \theta_{{S_{j} }} } \right)}}$ and $E_{{I_{j} }} = E_{0} e^{{i\left( {k_{{I_{j} }} r - 2\pi f_{{I_{j} }} t + \theta_{{I_{j} }} } \right)}}$. The photon pair generation rate and bandwidth in SPDC can be controlled by adjusting the pump power and spectral filter. In general, the photon detection rate by a single photon detector module is far less than MHz. Considering a detection module speed faster than GHz, consecutive photon pairs are treated independently throughout the coincidence measurement process. The coherent property of each generated photon pair is determined by Heisenberg’s uncertainty principle in terms of the energy-time relation: $\Delta f\Delta t \ge 1$. For a typical THz bandwidth $\Delta f$, the coherence time $\Delta t$ is on the order of ps. Compared with the corresponding coherence length $l_{C} \sim 100\;\upmu {\text{m}}$, the original wavelength $\lambda_{0}$ $\left( {\sim 0.5\;\upmu {\text{m}}} \right)$ of the pump is several orders of magnitude shorter than $l_{C}$. In other words, the $g^{\left( 1 \right)}$ correlation is much more sensitive than the $g^{\left( 2 \right)}$ correlation, where $g^{\left( 2 \right)}$ stands for a HOM dip.

According to the energy conservation law, the signal and idler photons in each pair are symmetrically detuned by $\pm \delta f_{j}$ from the half-frequency $\left( {f_{0} /2} \right)$ of the pump laser as shown in Fig. 1a. Due to spontaneous emission processes, however, the frequencies $f_{{S_{j} }}$ and $f_{{I_{j} }}$ of the jth photon pair are random within the bandwidth $\Delta f$. Similarly, the initial phases $\theta_{{S_{j} }}$ and $\theta_{{I_{j} }}$ are not determined, either. As analyzed, however, the difference phase $\delta \theta_{j}$ between $\theta_{{S_{j} }}$ and $\theta_{{I_{j} }}$ is fixed at $\uppi /2$^20^. This fact is also derived differently below in Fig. 1. Figure 1b originates from Ref.^24^ and is used to understand important quantum features. The first (second) MZI in Fig. 1b is controlled by $\Delta L_{1} \left( {\Delta L_{2} } \right)$, where $\zeta_{j} = \frac{2\pi }{{\lambda_{j} }}\Delta L_{1}$ $\left( {\upvarphi_{j} = \frac{2\pi }{{\lambda_{j} }}\Delta L_{2} } \right)$, and $\lambda_{j}$ is the wavelength of the jth photon. Regardless of nondegeneracy in $\upchi ^{\left( 2 \right)}$, all pairs are symmetrically detuned, whose corresponding phase difference is $\pm \delta f_{j} \tau = \pm \zeta_{j}$, where $\tau$ is the relative delay between paired photons for measurements.

The coincidence measurements between output ports α and β on a beam splitter BS1 are for the second-order intensity correlation $g^{\left( 2 \right)} \left(\uptau \right)$, where the jth output intensities are as follows (see Fig. S1 of the Supplementary Information):

1 $$I_{\alpha }^{j} \left( {r,t} \right) = I_{0} \left[ {1 + sin\left( {\upzeta _{j}^{\prime } } \right)} \right],$$

2 $$I_{\beta }^{j} \left( {r,t} \right) = I_{0} \left[ {1 - sin\left( {\upzeta _{j}^{\prime } } \right)} \right].$$

Phase $\upzeta _{j}^{\prime }$ is described as:

3 $$\upzeta _{j}^{\prime } \left( {r,t} \right) = \left( {\frac{{k_{0} }}{2} - \delta k_{j} } \right)\Delta L_{1} - \left( {\frac{{\omega_{0} }}{2} - \delta \omega_{j} } \right)\tau + \delta \varphi_{j} - 2\left( {\delta k_{j} r_{s} - \delta \omega_{j} t_{s} } \right).$$

For all $\delta f_{j}$-dependent photon pairs, $I_{\alpha } = \sum\nolimits_{j} {I_{\alpha }^{j} }$ and $I_{\beta } = \sum\nolimits_{j} {I_{\beta }^{j} }$. Equation (3) represents four different sources of the induced phase $\upzeta _{j}^{\prime }$. The first, $\left( {\frac{{k_{0} }}{2}\Delta L_{1} } \right)$, is a center frequency-related fundamental oscillation as a function of ${\Delta }L_{1}$: $2\lambda_{0}$-dependent fast oscillation. The second, $\left( {\delta k_{j} \Delta L_{1} } \right)$, is the detuning-caused slow oscillation, resulting in $\Delta f^{ - 1} \left( \tau \right)$-dependent decoherence. The third, $\left( {\delta \varphi_{j} } \right)$, is for a fixed relative phase $\uppi /2$ between the signal and idler photons in each pair. The last, $\left( {2\delta k_{j} r_{s} } \right)$, is for $\Delta L_{1}$-independent frequency beating between the paired photons, resulting in a fixed phase. Because of the wide spectrum in Fig. 1a, this beating results in a $\Delta f^{ - 1} \left( \tau \right)$-dependent wide envelope. Thus, Eq. (3) becomes a function of $\Delta L_{1}$ (or $\uptau$) only. However, all $\updelta f_{j}$-caused phase factors in Eq. (3) cancel each other out due to the $\pm\updelta f_{j}$ distribution of all photon pairs except for the fixed $\delta \varphi_{j}$ at coincidence detection. Thus, the mean values of the output intensities are uniform, resulting in $\left\langle {I_{\alpha } } \right\rangle = \left\langle {I_{0} } \right\rangle = I_{0}$ because $\left\langle {sin\left( {\upzeta _{j}^{\prime } } \right)} \right\rangle = 0$, where the signal and idler photons are interchangeable. This is the physical origin why there is no $g^{\left( 1 \right)}$ correlation in $g^{\left( 2 \right)} \left( \tau \right)$ in the first MZI. As analyzed for the second MZI below, this is also the physical origin of how $g^{\left( 1 \right)}$ is retrieved in $g^{\left( 2 \right)} \left( \tau \right)$ as previously observed^24^. By the way, in Ref.^24^, a typical HOM dip without $g^{\left( 1 \right)}$ fringe is observed in the first MZI, while the λ-dependent $g^{\left( 2 \right)}$ correlation for PBW has been observed in the second MZI. The observed PBWs in the second MZI, however, have been interpreted as the result of the HOM dip in the first MZI, which is contradictory to the present analysis.

According to the definition of intensity correlation $g_{\alpha \beta }^{\left( 2 \right)} \left( \tau \right)$ = $\frac{{\left\langle {I_{\alpha } I_{\beta } } \right\rangle }}{{\left\langle {I_{\alpha } } \right\rangle \left\langle {I_{\beta } } \right\rangle }}$, the following equation results from Eqs. (1) and (2):

4 $$g_{{\alpha \beta_{j} }}^{\left( 2 \right)} \left( {\tau ,\delta f_{j} } \right) = cos^{2} \left( {\upzeta _{j}^{\prime } } \right).$$

**Figure 2:**
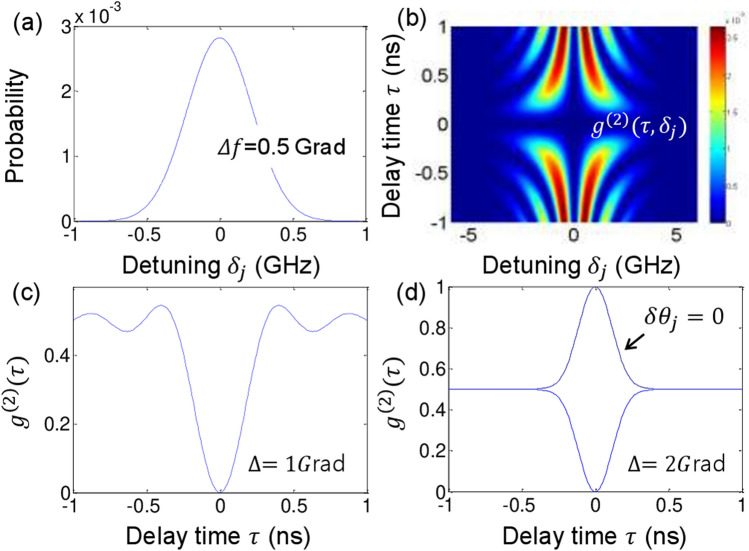
Numerical simulations of the intensity correlation in a typical HOM dip with $\delta \theta_{j} = \pm \pi /2$. (**a**) Photon distribution. (**b**) τ versus $\delta_{j}$. (**c**) and (**d**) Sum $g^{\left( 2 \right)} \left( \tau \right)$ for all $\delta_{j}$ for different coverage $\Delta f$. Dotted: $\delta \theta_{j} = 0$.

To satisfy anticorrelation of $g_{{\alpha \beta_{j} }}^{\left( 2 \right)} \left( {\tau = 0,\delta f_{j} } \right) = 0$ in a SPDC-based HOM dip, $\delta \theta_{j} = \pm \pi /2$ must be satisfied for each photon pair^20^. If $\uptau \ne 0$, Eq. (4) gradually decays and shows a typical HOM dip curve as a function of delay time $\uptau _{c} \left( { = \Delta f^{ - 1} } \right)$, where decay time $\uptau _{c}$ in $g^{\left( 2 \right)} \left( \tau \right) \left[ { = \sum\nolimits_{j} {g_{j}^{\left( 2 \right)} \left( {\tau ,\delta_{j} } \right)} } \right]$ is preset according to the inverse of the SPDC-generated photon bandwidth $\Delta f$, as shown in Fig. 2.

Figure 2 shows numerical calculations for Eq. (4). Figure 2a shows the Gaussian distribution of SPDC-generated photon pairs with the bandwidth of $\Delta f = 0.5 \times 10^{9}$ radians. According to Fig. 1a, the jth photon pair has different detuning at $\updelta f_{j}$, whose corresponding $g^{\left( 2 \right)} (\tau ,\delta f_{j}$) is shown in Fig. 2b. In Eq. (4), the jth photon pair must contribute to different $g^{\left( 2 \right)} \left( \tau \right)$ only because of the detuning dependent ${\upzeta }_{j}^{^{\prime}}$. By definition, $g^{\left( 2 \right)} \left( \tau \right)$ is obtained by averaging all $\delta f_{j}$-dependent coincidence measurements for a fixed τ. As shown in Fig. 2c,d, the maximum $g^{\left( 2 \right)} \left( \tau \right)$ is bound to $g^{\left( 2 \right)} \left( \tau \right) = 0.5$, where $g^{\left( 2 \right)} \left( \tau \right) = 0.5$ is a classical lower bound^20^. This upper limit of $g^{\left( 2 \right)} \left( \tau \right) = 0.5$ strongly supports the nonclassical phenomenon of entangled photon pairs^24^. If all of the spectral photon pairs are not fully covered for the measurements via spectral filtering, there is a wiggle in $g^{\left( 2 \right)} \left( \tau \right)$ as shown in Fig. 2c. This wiggle is due to incomplete coherence washout in the summation process^24^. Disappearance of the $g^{\left( 1 \right)}$ fringe in a HOM dip is not due to the measurement process or artifacts, but instead due to the inherent properties of the symmetrically detuned photon pairs in SPDC. If there is no relative phase between signal and idler photons $\left( {\updelta \theta_{j} = 0} \right)$, then there is no nonclassical feature in $g^{\left( 2 \right)} \left( \tau \right)$ as indicated by the dotted curve in Fig. 2d. If the relative phase $\delta \theta_{j}$ is random for all pairs, $g^{\left( 2 \right)} \left( \tau \right) = 1/2$ regardless of τ, representing the property of the individual particle ensemble^20^.

In the second MZI in Fig. 1b, the ${\Delta }L_{2}$ effect can be classified for bunched photons only on BS1 if ${\Delta }L_{1} \sim 0$. According to Eq. (3), all other terms become zero except for $\updelta \varphi_{j}$, which is $\uppi /2$ for all j. The bunched photons in each path of the MZI are composed of signal and idler photon pairs, whose detuning is exactly opposite across the center frequency $f_{0} /2$ as sown in Fig. 1a. Thus, whenever a nonzero ${\Delta }L_{2}$ occurs, the detuning $\updelta f_{j}$-caused phase terms in Eq. (3) are cancelled automatically due to the $+ / -$ relation in $\updelta f_{j}$. As a result, only the original $2\lambda_{0}$-dependent fast oscillation survives in the output fields. This is the unspoken secretes in the SPDC-based $g^{\left( 1 \right)}$ features observed in Ref.^24^ for $g^{\left( 2 \right)}$ measurements.

In the second MZI of Fig. 1b, the following amplitude relations are obtained for the final outputs ${\text{E}}_{A}$ and ${\text{E}}_{B}$:

5 $$\left[ {\begin{array}{*{20}c} {E_{A} } \\ {E_{B} } \\ \end{array} } \right]_{j} = \frac{1}{2}\left[ {\begin{array}{*{20}c} {1 - e^{{i\varphi_{j} }} } & {ie^{{i\zeta_{j}^{^{\prime}} }} \left( {1 + e^{{i\varphi_{j} }} } \right)} \\ {i\left( {1 + e^{{i\varphi_{j} }} } \right)} & { - e^{{i\zeta_{j}^{^{\prime}} }} \left( {1 - e^{{i\varphi_{j} }} } \right)} \\ \end{array} } \right]\left[ {\begin{array}{*{20}c} {E_{S} } \\ {E_{I} } \\ \end{array} } \right]_{j} .$$

From Eq. (5), the corresponding intensities are as follows (see Fig. S2 of the Supplementary Information):

6 $$I_{A}^{j} = I_{0} \left( {1 - cos\zeta_{j}^{\prime } \sin \varphi_{j} } \right),$$

7 $$I_{B}^{j} = I_{0} \left( {1 + cos\zeta_{j}^{\prime } \sin \varphi_{j} } \right)$$

The anticorrelation condition $\zeta_{j}^{\prime } = \pm \pi /2$ in Eq. (3), however, results in independence of $\varphi_{j}$. If $\zeta_{j}^{\prime } = 0$, $I_{A}^{j} = I_{0} \left( {1 - \sin \varphi_{j} } \right)$ and $I_{B}^{j} = I_{0} \left( {1 + \sin \varphi_{j} } \right)$ are obtained. In this case, however, the photon bunching or anticorrelation in Eqs. (3) and (4) is violated, resulting in the classical feature of $g_{{\alpha \beta_{j} }}^{\left( 2 \right)} \left( {\tau ,\delta_{j} } \right) = 1$ from Eq. (4) (see Fig. S3 of the Supplementary Information). Although the normalized coincidence detection measurement becomes $R_{AB}^{j} = \frac{1}{2}\left( {1 + cos2\varphi_{j} } \right)$, $g_{{AB_{j} }}^{\left( 2 \right)} \left( {\tau_{\zeta } ,\tau_{\varphi } ,\delta_{j} } \right) = 1$ shows a classical feature. The $cos2\varphi_{j}$ modulation term in $R_{AB}$ is a typical classical feature of the intensity product from a single MZI, satisfying $g_{{\alpha \beta_{j} }}^{\left( 2 \right)} \left( {\tau ,\delta_{j} } \right) = 0$ for photon bunching in a HOM dip violates $R_{AB}^{j} = \frac{1}{2}\left( {1 + cos2\varphi_{j} } \right)$ (see Fig. S3 of the Supplementary Information). Thus, the observations of $cos2\varphi_{j}$ modulation in Ref.^24^ are not from simultaneous satisfaction of nonclassical features in both MZIs of Fig. 1b. For ${\text{N}} \ge 4$ in PBW, an inter-MZI superposition scheme can be a quantum solution as proposed^21,22^ and demonstrated^23^. Otherwise, an intrinsically multi-photon entangled photon pair must be involved with violation of HOM dip in the first MZI.

**Figure 3:**
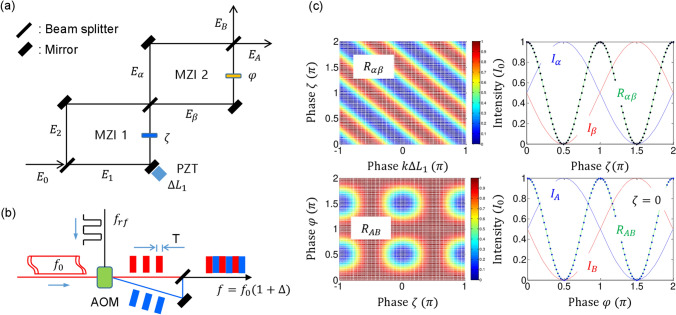
Schematic of deterministic entangled photon-pair generations. (**a**) A coupled MZI structure. (**b**) Basis randomness for $\upzeta \left( {0;\uppi } \right)$. (**c**) Numerical calculations for $R_{ij} = I_{i} I_{j} *4$ at ${\text{k}}\Delta L_{1} = \frac{\pi }{2}$. (Top row) For $I_{\alpha }$ and $I_{\beta }$. (bottom row) For $I_{A}$ and $I_{B}$. $I_{i}$ and $I_{j}$ are interchangeable on behalf of AOM.

Figure 3 shows a coherence version of the entangled photon-pair generation comparable with Fig. 1. Because MZI works for either a single photon or coherence light equivalently^26^, there is no difference in the photon characteristics. The photons propagating along different paths of MZI 1 are strongly coupled by the relative phase of $$\uppi /2$$ created from the first BS, regardless of the input photon’s wavelength^27^. The matrix representations for Fig. 3a are as follows without considering $$\Delta L_{1}$$: $$I_{\alpha } = \frac{{I_{0} }}{2}\left( {1 - cos\zeta } \right)$$, $$I_{\beta } = \frac{{I_{0} }}{2}\left( {1 + cos\zeta } \right)$$, $$I_{A} = \frac{{I_{0} }}{2}\left[ {1 - sin\varphi sin\zeta } \right]$$, and $$I_{B} = \frac{{I_{0} }}{2}\left[ {1 + sin\varphi sin\zeta } \right]$$ (see Fig. S4 of the Supplementary Information). Using an acousto-optic modulator (AOM) driven by an RF pulse generator with an RF frequency of $$f_{rf}$$, the role of $$\updelta f_{j}$$-caused random phases in Fig. 1 can be satisfied by a 50% duty cycle of AOM between 0 and $$f_{rf}$$, as shown in Fig. 3b. In other words, the zeroth (original $$f_{0}$$) and first-order ($$f_{0} + f_{rf} T)$$ diffracted light pulses are combined, where T is the RF pulse duration. If $$2{\uppi }f_{rf} T = \pi$$, the output direction is reversed. Thus, the average of each output intensity is uniform, $$I_{\alpha } = I_{\beta } = I_{A} = I_{B} = I_{0}$$, satisfying randomness. Including the $${\Delta }L_{1}$$ effect in $$\upzeta$$, the revised output intensities are as follows:

8 $$I_{\alpha } = \frac{{I_{0} }}{2}\left( {1 - cos\zeta^{\prime } } \right),$$

9 $$I_{\beta } = \frac{{I_{0} }}{2}\left( {1 + cos\zeta^{\prime } } \right),$$

10 $$I_{A} = \frac{{I_{0} }}{2}\left[ {1 - sin\varphi sin\zeta^{\prime } } \right],$$

11 $$I_{B} = \frac{{I_{0} }}{2}\left[ {1 + sin\varphi sin\zeta^{\prime } } \right],$$

where $$\upzeta ^{\prime } = \zeta + k\Delta L_{1} \left( {2\pi f_{rf} T} \right)$$.

Figure 3c shows numerical calculations for Eqs. (8)–(11) (see also Figs. S4 and S5 of the Supplementary Information). For $\upzeta ^{\prime } = \zeta + \pi /2$, Eqs. (8)–(11) are rewritten as $I_{\alpha } = \frac{{I_{0} }}{2}\left( {1 + sin\zeta } \right)$, $I_{\beta } = \frac{{I_{0} }}{2}\left( {1 - sin\zeta } \right)$, $I_{A} = \frac{{I_{0} }}{2}\left( {1 + sin\varphi {\text{cos}}\zeta } \right)$, and $I_{B} = \frac{{I_{0} }}{2}\left( {1 - sin\varphi {\text{cos}}\zeta } \right)$. The normalized intensity product $R_{ij}$ between $I_{i}$ and $I_{j}$ is the same as $g_{\alpha \beta }^{\left( 2 \right)} \left( \zeta \right) = \frac{1}{2}\left( {1 - cos2\zeta } \right)$ for MZI 1 and $g_{AB}^{\left( 2 \right)} \left( \varphi \right) = \frac{1}{2}\left( {1 - sin^{2} \varphi sin^{2} \zeta } \right)$ for MZI 2 due to the randomness by AOM. To satisfy the anticorrelation condition for $g_{\alpha \beta }^{\left( 2 \right)} \left( \zeta \right)$, $\zeta = \pm \pi /2$ is obtained as shown in the top panels of Fig. 3c. For the same conditions of $\zeta = \pm \pi /2$, however, there is no way to satisfy the quantum feature between $I_{A}$ and $I_{B}$, unless ${\Delta }L_{1}$ is changed. For $R_{AB} = 0$, $\zeta = \pm n\pi$ must be satisfied as shown in the bottom panels, where n = 0,1,2… As analyzed in Fig. 2, this also proves the violation of the quantum feature analysis in Ref.^24^. In a short summary, the correct condition for the quantum feature generation in Fig. 3a for the final outputs is to break the anticorrelation condition in $\upzeta$. Neither way, the PBW cannot be possible in the directly coupled MZI scheme due to this reason, where Fig. 3c is just for the diffraction limit of the Rayleigh criterion in the intensity product: $R_{AB} = \left( {1 + cos2\varphi } \right)/2$. As presented elsewhere, such PBW can be achieved by CBW via path superposition^28^. For this, an intermediate dummy MZI must be inserted between two MZIs in Fig. 3a.

## Conclusion

In conclusion, the quantum features of anticorrelation and PBW were analyzed in a doubly coupled MZI system using pure coherence optics, where SPDC-generated symmetrically distributed entangled photon pairs played an essential role in both $g^{\left( 1 \right)}$ disappearance in the first MZI and the $g^{\left( 1 \right)}$ retrieval in the second MZI. Based on the $\chi^{\left( 2 \right)}$-generated entangled photon-pair distribution, the relative $\uppi /2$ phase difference between all paired photons was derived as an essential condition for anticorrelation, i.e. a HOM dip. Moreover, the anticorrelation condition in the first MZI violated quantum feature generation conditions in the second MZI. In other words, satisfying the anticorrelation in one MZI resulted in destruction of quantum features in the other MZI. As a result, PBW could not be generated from the doubly coupled MZI system if a HOM dip condition is satisfied simultaneously. Instead, quantum superposition between MZIs via a dummy MZI can be used to create PBW^21–23,28^. Otherwise, a single MZI is available for PBW only with preset higher-order entangled photon pairs such as a N00N state via an action of quantum operators for a consecutive measurement process according to the particle nature of quantum mechanics^29^. Finally, a deterministic coherence version of entangled light pair generation was proposed and analyzed using pure coherence optics applicable to both single photons and coherent light without violation of quantum mechanics.

## Methods

The numerical calculations in Figs. 2 and 3 were performed by MATLAB using the equations in the text. The data that support the findings of this study are available from the corresponding author upon reasonable request.

## Supplementary information

Supplementary Information.
