# Supplementary material for: Coherently controlled quantum features in a coupled interferometric scheme
Source: Sci Rep. 2021 May 27;11:11188. doi: 10.1038/s41598-021-90668-8 (PMC8159952; doi:10.1038/s41598-021-90668-8)
Supplement: Supplementary file 1 — Supplementary Information. [file 41598_2021_90668_MOESM1_ESM.pdf]

Supplementary Information

Coherently controlled quantum features in a coupled interferometric scheme

B. S. Ham
GIST

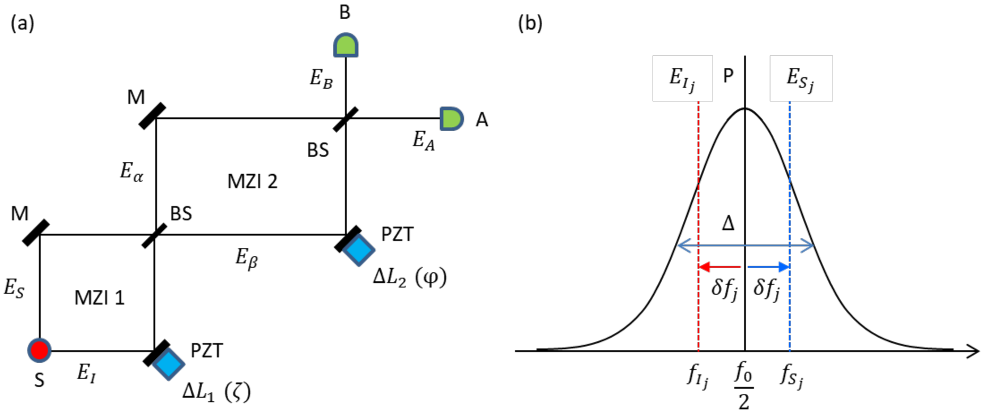

Fig. S1. Schematic of quantum correlation. (a) S: SPDC light source, M: mirror, BS: beam splitter, PZT: Piezo-electric transducer, A/B: photon detectors. (b) SPDC-generated photon bandwidth. Δ, bandwidth, E_{S_j}/E_{I_j}: j[th] signal/idler photon pair with related symmetric frequency detuning $\delta f_{S_j}/\delta f_{I_j}$. f_0: pump frequency.

Figure S1(a) shows a schematic diagram of quantum feature generation using entangled photon pairs E_{S_j} and E_{I_j} by spontaneous parametric down conversion (SPDC) process in a $\chi^{(2)}$ nonlinear optical medium, where E_{S_j} and E_{I_j} represent the jth signal and idler photon pair. The PZT controlled path-length changes by ΔL_1 and ΔL_2 in Fig. 1(a) indicate relative path-length differences between two paths in each MZI. The jth signal and idler photon pair (E_{S_j}, E_{I_j}) is randomly distributed with $\pm\delta f_j$ across the center frequency of $f_0/2$ as shown in Fig. 1(b), were f_0 is the pump frequency used for SPDC. In a pure classical picture of coherence optics, the jth photon pair is described by:

$$E_{S_j}(r,t) = E_0 e^{i\left(k_{S_j} r_S - \omega_{S_j} t_S + \varphi_{S_j}\right)}$$
$$= E_0 e^{i\left[\left(\frac{k_0}{2}+\delta k_j\right) r_S - \left(\frac{\omega_0}{2}+\delta\omega_j\right) t_S + \varphi_{S_j}\right]}. \quad \text{(S1)}$$

$$E_{I_j}(r,t) = E_0 e^{i\left(k_{I_j} r_I - \omega_{I_j} t_I + \varphi_{I_j}\right)}$$
$$= E_0 e^{i\left[\left(\frac{k_0}{2}-\delta k_j\right)(r_S+\Delta L_1) - \left(\frac{\omega_0}{2}-\delta\omega_j\right)(t_S+\tau) + \varphi_{I_j}\right]}$$
$$= E_0 e^{i\left[\left(\frac{k_0}{2}+\delta k_j\right) r_S - \left(\frac{\omega_0}{2}+\delta\omega_j\right) t_S + \varphi_{S_j}\right]} e^{i\left[\left(\frac{k_0}{2}-\delta k_j\right)\Delta L_1 - \left(\frac{\omega_0}{2}-\delta\omega_j\right)\tau + \delta\varphi_j - 2\left(\delta k_j r_S - \delta\omega_j t_S\right)\right]}, \quad \text{(S2)}$$

where $\delta k_j = k_{I_j} - k_{S_j}$, $\delta\omega_j = \omega_{I_j} - \omega_{S_j}$, $\delta\varphi_j = \varphi_{I_j} - \varphi_{S_j}$, $\tau_1 = \Delta L_1/c$, and c is the speed of light. The subscript j represents for the jth photon in Fig. 1(b) determined by δf_j $(= \frac{\delta\omega_j}{2\pi})$. All entangled photon pairs of E_{S_j} and E_{I_j} are symmetrically detuned across the half pump frequency $f_0/2$ to satisfy the energy conservation law in the $\chi^{(2)}$ process (see Fig. S1(b)). Here, φ_{S_j} and φ_{I_j} are initial phases randomly assigned to the jth signal and idler photon pair, respectively. As derived in the main text, the relative phase $\delta\varphi_j$ in each pair is always fixed at $\pi/2$. A typical SPDC light source has a wide bandwidth $\Delta > 10$ nm or > 5 THz at $\lambda = 800$ nm. Equation (S2) can be rewritten as:

$$E_{I_j}(r,t) = E_S e^{i\left[\left(\frac{k_0}{2}-\delta k_j\right)\Delta L_1 - \left(\frac{\omega_0}{2}-\delta\omega_j\right)\tau + \delta\varphi_j - 2\left(\delta k_j r_S - \delta\omega_j t_S\right)\right]}, \quad \text{(S3)}$$

The exponent in equation (S3) indicates a relative phase $\zeta_j{}'$ between the paired photons in MZI 1 when impinged on the first BS:

$$\zeta_j{}'(r,t) = \left(\frac{k_0}{2} - \delta k_j\right)\Delta L_1 - \left(\frac{\omega_0}{2} - \delta\omega_j\right)\tau + \delta\varphi_j - 2(\delta k_j r_s - \delta\omega_j t_s). \quad \text{(S4)}$$

In MZI 1, the following matrix representations are obtained for Fig. S1:

$$\begin{bmatrix} E_\alpha \\ E_\beta \end{bmatrix} = [BS][Z]\begin{bmatrix} E_I \\ E_S \end{bmatrix}$$
$$= \frac{E_S}{\sqrt{2}}\begin{bmatrix} 1 & i \\ i & 1 \end{bmatrix}\begin{bmatrix} e^{i\zeta'} & 0 \\ 0 & 1 \end{bmatrix}\begin{bmatrix} 1 \\ 1 \end{bmatrix}$$
$$= \frac{E_S}{\sqrt{2}}\begin{bmatrix} e^{i\zeta'} & i \\ ie^{i\zeta'} & 1 \end{bmatrix}\begin{bmatrix} 1 \\ 1 \end{bmatrix}, \quad \text{(S5)}$$

where $[BS]$ is the BS matrix, and $[Z]$ is the ΔL_1 −caused phase matrix. Thus,

$$E_\alpha = \frac{E_S}{\sqrt{2}}(e^{i\zeta'} + i), \quad \text{(S6)}$$

$$E_\beta = \frac{iE_S}{\sqrt{2}}(e^{i\zeta'} - i). \quad \text{(S7)}$$

The corresponding intensities are as follows:

$$I_\alpha = I_0(1 + sin\zeta'), \quad \text{(S8)}$$

$$I_\beta = I_0(1 - sin\zeta'). \quad \text{(S9)}$$

The phase ζ' in equations S(8) and S(9) is for $\zeta_j{}'$ in equation (S4), where $\langle sin\zeta' \rangle = 0$ due to $\pm\zeta'$. Thus, the average value of $\langle I_\alpha \rangle$ and $\langle I_\beta \rangle$ turns out to be $sin\zeta'$ as a function of ΔL_1. Once again, the δf_j −caused phase in ζ' symmetrically spans both + and − regions across $f_0/2$ in Fig. S1(b), resulting in $\langle sin\zeta' \rangle = 0$ in average.

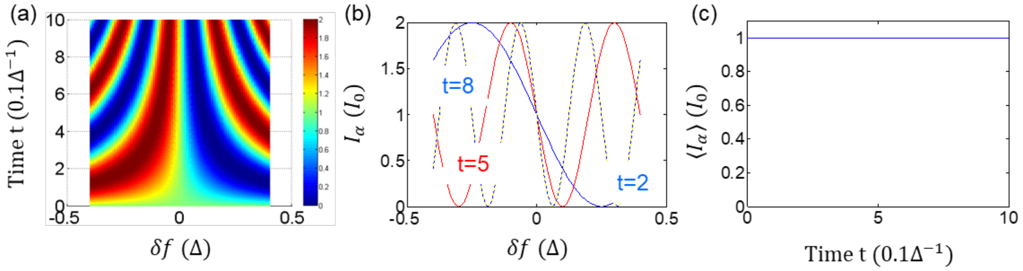

Fig. S2. Numerical simulations for equation (S8). Figure S2 is generated using MATLAB, version R2014a.

This symmetric relation in the photon pairs of SPDC process is the bedrock of quantum superposition, resulting in randomness. In other words, the bunched photon distribution at $\tau = 0$ in equation (S8) and (S9) is completely random. Thus, $\langle I_\alpha \rangle = \langle I_\beta \rangle = I_0$. As a result, the intensity correlation for the output intensities of MZI 1 results in a HOM dip:

$$g^{(2)}(\tau) = \langle 1 - sin^2(\zeta') \rangle. \quad \text{(S10)}$$

At the coincidence detection time $\tau = 0$ or $\Delta L_1 = 0$, equation (S10) is $g^{(2)}(\tau = 0) = 0$ due to $\zeta'(\tau = 0) = \delta\varphi_j = \pi/2$ in equation (S4). However, $g^{(2)}(\tau) = 1/2$ if $\tau \gg \Delta^{-1}$ due to $\langle sin^2(\zeta') \rangle = 1/2$. This is the case for completely individual particles, representing the classical lower bound. In equation (S4), the $2\lambda_0$ −dependent fast oscillation is buried in the Δ −dependent slow oscillation.

The matrix representation in MZI 2 is as follows:

$$\begin{bmatrix} E_A \\ E_B \end{bmatrix} = [BS][\Phi]\begin{bmatrix} E_\alpha \\ E_\beta \end{bmatrix}$$
$$= \frac{1}{\sqrt{2}}\begin{bmatrix} 1 & i \\ i & 1 \end{bmatrix}\begin{bmatrix} 1 & 0 \\ 0 & e^{i\varphi} \end{bmatrix}\begin{bmatrix} E_\alpha \\ E_\beta \end{bmatrix}$$
$$= \frac{E_S}{2}\begin{bmatrix} 1 & ie^{i\varphi} \\ i & e^{i\varphi} \end{bmatrix}\begin{bmatrix} e^{i\zeta'} & i \\ ie^{i\zeta'} & 1 \end{bmatrix}\begin{bmatrix} 1 \\ 1 \end{bmatrix}$$
$$= \frac{E_S}{2}\begin{bmatrix} e^{i\zeta'}(1 - e^{i\varphi}) & i(1 + e^{i\varphi}) \\ ie^{i\zeta'}(1 + e^{i\varphi}) & -(1 - e^{i\varphi}) \end{bmatrix}\begin{bmatrix} 1 \\ 1 \end{bmatrix}. \quad \text{(S11)}$$

From equation (S11), the following relations are obtained:

$$E_A = \frac{iE_S}{2}\left[e^{i\zeta'}(1 - e^{i\varphi}) + i(1 + e^{i\varphi})\right], \quad \text{(S12)}$$

$$E_B = \frac{iE_S}{2}\left[e^{i\zeta'}(1 + e^{i\varphi}) + i(1 - e^{i\varphi})\right], \quad \text{(S13)}$$

$e^{i\zeta'} = i$. Thus, the corresponding intensities are as follows:

$$I_A = \frac{I_0}{4}\left[e^{i\zeta'}(1 - e^{i\varphi}) + i(1 + e^{i\varphi})\right]\left[e^{-i\zeta'}(1 - e^{-i\varphi}) - i(1 + e^{-i\varphi})\right]$$

$$= \frac{I_0}{4}\left\{(1 - e^{i\varphi})(1 - e^{-i\varphi}) + (1 + e^{i\varphi})(1 + e^{-i\varphi}) + i\left[(1 + e^{i\varphi})(1 - e^{-i\varphi})e^{-i\zeta'} - (1 - e^{i\varphi})(1 + e^{-i\varphi})e^{i\zeta'}\right]\right\}$$

$$= \frac{I_0}{4}\left[4 - 2sin\varphi\left(e^{-i\zeta'} + e^{i\zeta'}\right)\right]$$

$$= I_0[1 - sin\varphi cos\zeta']. \quad \text{(S13)}$$

Likewise,

$$I_B = I_0[1 + sin\varphi cos\zeta']. \quad \text{(S14)}$$

Here, the photon bunching condition is $\zeta' = \pm\pi/2$, as resulted from equation (S4). The average value of $\langle I_A \rangle = \langle I_B \rangle = I_0$ is satisfied regardless of φ due to $\zeta' = \pi/2$. Then, there is no φ −dependent modulation in the coincidence measurements. To make it work for the nonclassical feature of PBW, $\zeta' = 0$ must be satisfied, resulting in $I_A = I_0(1 - sin\varphi)$ and $I_B = I_0(1 + sin\varphi)$. Then, the normalized coincidence detection rate becomes:

$$R_{AB} = 1 - sin\varphi^2$$

$$= \frac{1}{2}(1 + cos2\varphi). \quad \text{(S15)}$$

However, equation (S15) is not nonclassical or PBW, but shows the classical diffraction limit such as in a single MZI. The condition of PBW in the output intensity should be $\frac{1}{2}(1 + cos4\varphi)$ as shown in ref. 12. Only possible case for this is biphoton interactions on a BS, resulting from bunched photons in both paths.

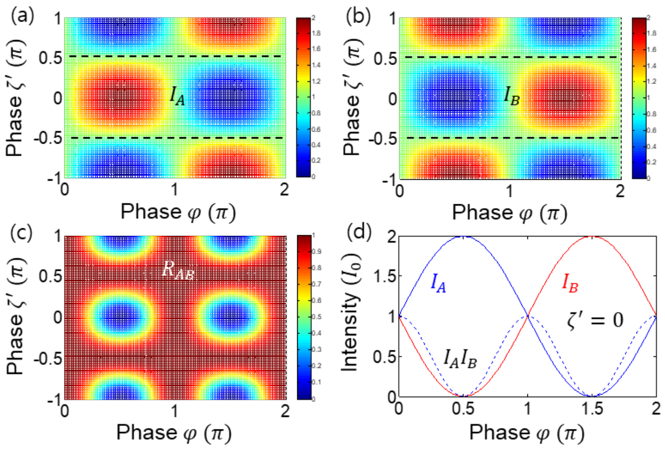

Fig. S3. Numerical simulations for Fig. 1. (a) and (b) Intensities I_A in path A and I_B in path B. (c) coincidence detection rate R_{AB}. (d) Output intensity for $\zeta = 0$. Dotted: R_{AB}. $\zeta' = \zeta + \pi/2$. Figure S3 is generated using MATLAB, version R2014a.

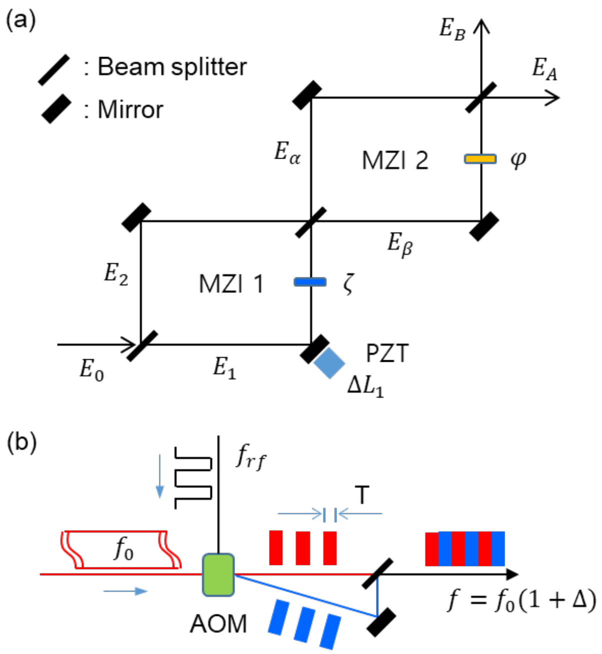

Fig. S4. A coherence version of entangled light pair generation. (a) Schematic of macroscopic quantum feature generation. (b) Pulse sequence for basis randomness. ΔL_1 is preset to be $\pi/2$ in phase difference between E_1 and E_2 without ζ.

Figure S4 is for Fig. 3 in the main text using coherent input light E_0. Using matrix representation, the following relations are obtained:

$$\begin{bmatrix} E_A \\ E_B \end{bmatrix} = [BS][\Phi][BS][Z][BS]\begin{bmatrix} E_0 \\ 0 \end{bmatrix}$$
$$= \frac{1}{2\sqrt{2}}\begin{bmatrix} 1 & i \\ i & 1 \end{bmatrix}\begin{bmatrix} 1 & 0 \\ 0 & e^{i\varphi} \end{bmatrix}\begin{bmatrix} 1 & i \\ i & 1 \end{bmatrix}\begin{bmatrix} e^{i\zeta} & 0 \\ 0 & 1 \end{bmatrix}\begin{bmatrix} 1 & i \\ i & 1 \end{bmatrix}\begin{bmatrix} E_0 \\ 0 \end{bmatrix}$$
$$= \frac{1}{2\sqrt{2}}\begin{bmatrix} 1-e^{i\varphi} & i(1+e^{i\varphi}) \\ i(1+e^{i\varphi}) & -(1-e^{i\varphi}) \end{bmatrix}\begin{bmatrix} e^{i\zeta} & ie^{i\zeta} \\ i & 1 \end{bmatrix}\begin{bmatrix} E_0 \\ 0 \end{bmatrix}$$
$$= \frac{1}{2\sqrt{2}}\begin{bmatrix} e^{i\zeta}(1-e^{i\varphi})-(1+e^{i\varphi}) & i[e^{i\zeta}(1-e^{i\varphi})+(1+e^{i\varphi})] \\ i[e^{i\zeta}(1+e^{i\varphi})-(1-e^{i\varphi})] & -e^{i\zeta}(1+e^{i\varphi})-(1-e^{i\varphi}) \end{bmatrix}\begin{bmatrix} E_0 \\ 0 \end{bmatrix}. \quad \text{(S16)}$$

Thus,

$$E_A = \frac{E_0}{2\sqrt{2}}[e^{i\zeta}(1-e^{i\varphi})-(1+e^{i\varphi})], \quad \text{(S17)}$$
$$E_B = \frac{iE_0}{2\sqrt{2}}[e^{i\zeta}(1+e^{i\varphi})-(1-e^{i\varphi})]. \quad \text{(S18)}$$

The corresponding intensities are as follows:

$$I_A = \frac{I_0}{8}[e^{i\zeta}(1-e^{i\varphi})-(1+e^{i\varphi})][e^{-i\zeta}(1-e^{-i\varphi})-(1+e^{-i\varphi})],$$
$$= \frac{I_0}{8}[(1-e^{i\varphi})(1-e^{-i\varphi})+(1+e^{i\varphi})(1+e^{-i\varphi})-e^{i\zeta}(1-e^{i\varphi})(1+e^{-i\varphi})-e^{-i\zeta}(1-e^{-i\varphi})(1+e^{i\varphi})],$$
$$= \frac{I_0}{2}(1-sin\varphi sin\zeta). \quad \text{(S19)}$$

Including the preset $\frac{\pi}{2}$ for ΔL_1,

$$I_A = \frac{I_0}{2}(1+sin\varphi cos\zeta). \quad \text{(S20)}$$

Likewise,

$$I_B = \frac{I_0}{2}(1-sin\varphi cos\zeta). \quad \text{(S21)}$$

For ζ ∈ {0, π}, $I_A = \frac{I_0}{2}(1 \pm sin\varphi)$ and $I_B = \frac{I_0}{2}(1 \mp sin\varphi)$. For the alternative modulations as shown in Fig. S4(b), the mean intensity values are $\langle I_A \rangle = \langle I_A \rangle = I_0$. Thus, intensity correlation between I_A and I_B is as follows:

$$g^{(2)}(\varphi) = 1 - sin\varphi^2$$
$$= \frac{1}{2}(1 + cos2\varphi). \qquad \text{(S22)}$$

For E_α and E_α,

$$\begin{bmatrix} E_\alpha \\ E_\beta \end{bmatrix} = \frac{1}{2}[\Phi][BS][\mathrm{Z}][BS]\begin{bmatrix} E_0 \\ 0 \end{bmatrix}$$
$$= \frac{1}{2}\begin{bmatrix} 1 & 0 \\ 0 & e^{i\varphi} \end{bmatrix}\begin{bmatrix} e^{i\zeta} - 1 & i(e^{i\zeta} + 1) \\ i(e^{i\zeta} + 1) & 1 - e^{i\zeta} \end{bmatrix}\begin{bmatrix} E_0 \\ 0 \end{bmatrix}$$
$$= \begin{bmatrix} e^{i\zeta} - 1 & i(e^{i\zeta} + 1) \\ ie^{i\varphi}(e^{i\zeta} + 1) & e^{i\varphi}(1 - e^{i\zeta}) \end{bmatrix}\begin{bmatrix} E_0 \\ 0 \end{bmatrix}. \qquad \text{(S23)}$$

Thus, the corresponding intensities are as follows:

$$I_\alpha = \frac{I_0}{2}(1 - cos\zeta). \qquad \text{(S24)}$$
$$I_\beta = \frac{I_0}{2}(1 + cos\zeta). \qquad \text{(S25)}$$

Including the preset $\frac{\pi}{2}$ for ΔL_1,

$$I_\alpha = \frac{I_0}{2}(1 - sin\zeta). \qquad \text{(S26)}$$
$$I_\beta = \frac{I_0}{2}(1 + sin\zeta). \qquad \text{(S27)}$$

For ζ ∈ {0, π}, $I_\alpha = I_\beta = \frac{I_0}{2}$. Thus, $\langle I_\alpha \rangle = \langle I_\beta \rangle = I_0/2$ is satisfied.

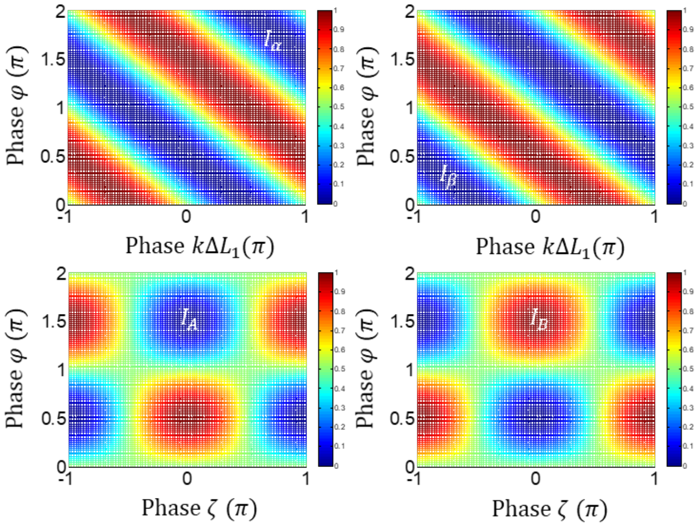

Fig. S5. Numerical calculations for equations. $k\Delta L_1 = \frac{\pi}{2}$. (Top row) For equations (S24) and S(25). (Bottom row) For equations (S20) and S(21). Figure S5 is generated using MATLAB, version R2014a.
